# Supplementary material for: A Global Screen for Assembly State Changes of the Mitotic Proteome by SEC-SWATH-MS
Source: Cell Syst. 2020 Feb 26;10(2):133–155.e6. doi: 10.1016/j.cels.2020.01.001 (PMC7042714; doi:10.1016/j.cels.2020.01.001)

O43353 | RIPK2_HUMAN | RIPK2 CARDIAK RICK RIP2 UNQ277/PRO314/PRO34092
Monomer MW [kDa]: 61.195 Monomer expected elution fraction: 44

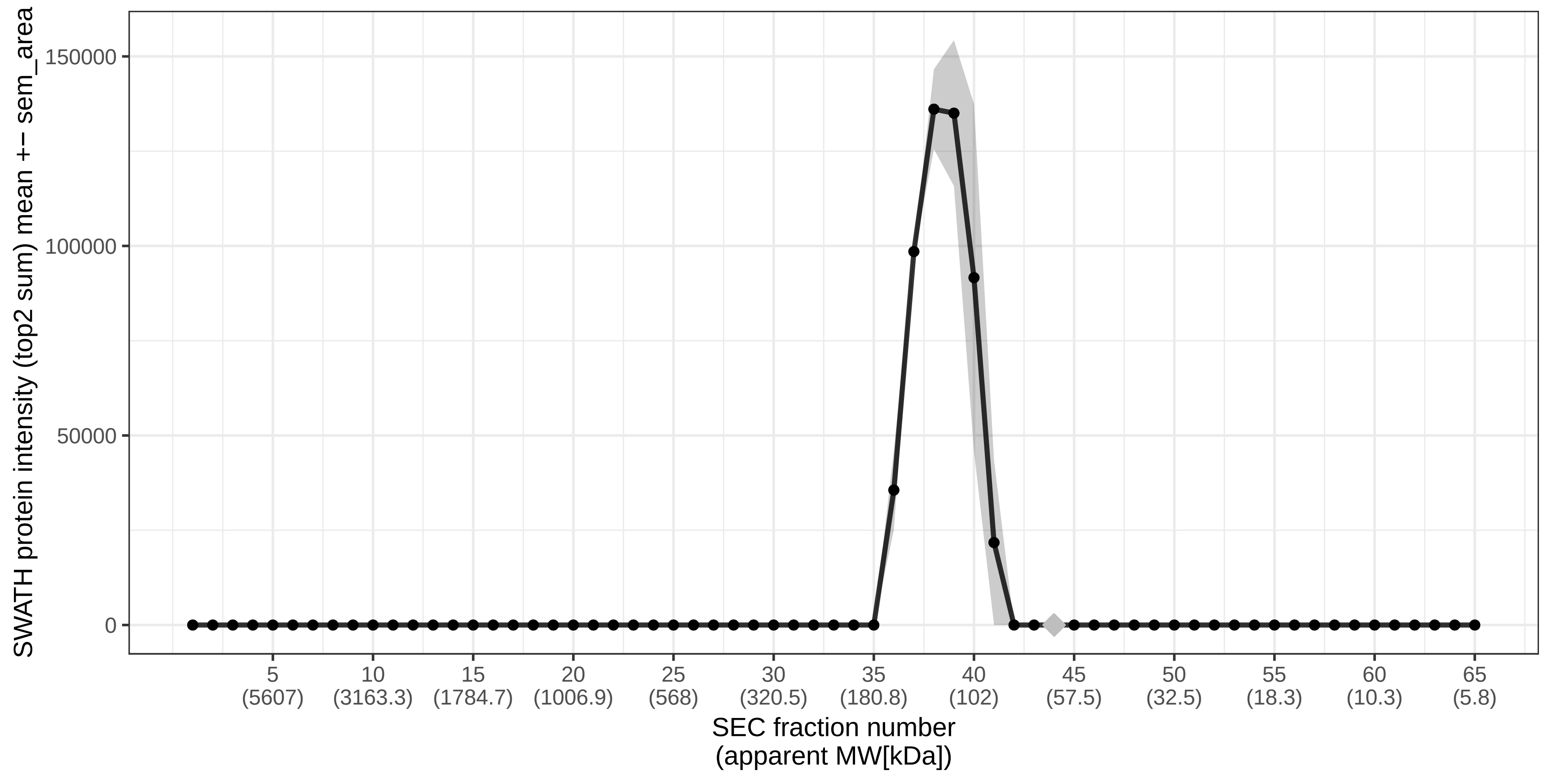

Supplement: Data S1. SEC-SWATH-MS Protein Chromatograms, Related to Figure 1 [file mmc6.zip › SECchrom_O43353_RIPK2_HUMAN_RIPK2_CARDIAK_RICK_RIP2_UNQ277_.pdf]
